# In vivo therapeutic potential of *Inula racemosa* in hepatic ischemia–reperfusion injury following orthotopic liver transplantation in male albino rats

**DOI:** 10.1186/s13568-017-0511-1

**Published:** 2017-11-22

**Authors:** Zhuoyi Wang, Lei Geng, Zhiyun Chen, Bingyi Lin, Mangli Zhang, Shusen Zheng

**Affiliations:** 1 Key Laboratory of Combined Multi-organ Transplantation, Ministry of Public Health, Key Laboratory of Organ Transplantation, Zhejiang, 310003 China; 2 Division of Hepatobiliary and Pancreatic Surgery, Department of Surgery, First Affiliated Hospital, School of Medicine, Zhejiang University, Zhejiang, 310003 China

**Keywords:** *Inula racemosa*, p53, ALT, Cytokines, Antioxidant

## Abstract

Hepatic ischemia–reperfusion (I/R) injury mainly occurs following hepatic resection and liver transplantation and cause severe liver damage, organ injuries, and dysfunction. Pro-inflammatory cytokines that promote injury are released when kupffer cell activates after getting induced by I/R. Repercussions of oxidative stress and cardiac function against isoproterenol based myocardial infarction are caused by flavonol glycosides which are found in high concentrations in *Inula racemosa* (Ir).The root was deemed to have analgesic and anti-inflammatory effects, and no report has been published about the liver-protective activity against hepatic I/R. Therefore, the present study was aimed to understand the therapeutic impact of Ir in hepatic I/R injury. Male albino, Wistar strain rats were used and were grouped into four total phenolic content, free radical scavenging activity and serum enzymes were determined. Histopathological and immunohistochemical analysis were also carried out. Inflammatory cytokines such as tumor necrosis factor-alpha (TNF-α) and interleukin (IL-6) and protein expression of p53, bax, and bcl-2 were determined. The administration of extracts of Ir significantly increased total phenolic and free radical scavenging activity. Altered cellular morphology, cytokines and aspartate aminotransferase (AST), alanine aminotransferase (ALT), alkaline phosphatase (ALP), and lactate dehydrogenase (LDH) were returned to near normal level. IL-6 and TNF-α levels were reduced more than 25% following treatment. Also, the protein expression of p53, bax, and bcl-2 were also returned to near normal level. Taking all these data together, it is suggested that the extracts of Ir may be a potential therapeutic agent for providing several beneficial effects in hepatic I/R injury.

## Introduction

When after a period of ischemia blood supply returns to the tissue causing tissue abrasion is reperfusion injury (Grace 2005). During the ischemic period due to the absence of oxygen and essential nutrients a condition occurs, rather than restoration of oxidative damage and inflammation through oxidative stress is caused as a result of the restoration of circulation. Liver ischemia–reperfusion (I/R) injury is well denoted as a notable reason for mortality and morbidity (Glantzounis et al. 2005). It often occurs in liver transplantation (Liu et al. 1991) and resections (Caldwell-Kenkel et al. 1991; Deschênes et al. 1998) where ischemic liver or anoxic injury takes place. It also occurs as a repercussion of hypoxia or insufficient perfusion occurring due to certain conditions that lower blood flow to the liver. Latter materialize in cardiogenic, hemorrhagic with fluid resuscitation (Yamakawa et al. 2000) in abdominal compartment syndromes (Okano et al. 2002) in cardiovascular and laparoscopic surgery (Glantzounis et al. 2001; Moore et al. 2005).

Liver transplantation, I/R injury is pertinent to the growth of primary graft dysfunction (occurrence in 10–25% of grafts) and primary graft non-function (an event in 5% of grafts) (Clavien et al. 2007). High rates of mortality are observed in both conditions. I/R injury elevate the occurrence of graft rejection (Fellstrom et al. 1998). Transplantation or liver resection with steatotic livers is another area where I/R injury affect. Some degree of liver steatosis has been observed in 25% of the western population (Selzner and Clavien 2001), the vast mass of TG inside the cytoplasm, ascribed to the effects of obesity, excess diabetes alcohol, and drugs.

Medicinal plants and phytochemicals have intensified because of potential chemotherapeutic values in animal diseases*. The root of Inula racemosa* (Ir) has been considered to exhibit cardio-protective effect and relieve ischemic pain (Manipuri et al. 2013). Sesquiterpenes, alloalantolactone, isoalantolactone, and alantolactone which are considered for therapeutic potential. Some glycosides, eudesmenes, germacranolides are also present in it. Ir should be considered for future studies as they offer new substitutes to the therapeutic options are very limited for liver diseases (Veteläinen et al. 2007; Kaplowitz 2000; Muthuviveganandavel et al. 2008; Olthoff et al. 2010; Gibson and Dudley 1984; Sylvia and Adam 1972). Several chemically defined molecules have been extracted from natural origins due to strong hepatoprotective activities epitomize an important source for effective liver protective agents. The need for this study exists on this basis.

## Materials and methods

### Chemicals

Xylazine, ketamine hydrochloride, chloroform, *n*-hexane, dimethyl sulfoxide (DMSO), and spirit were obtained from Sigma-Aldrich (USA). Aspartate aminotransferase (AST), alanine aminotransferase (ALT), alkaline phosphatase (ALP), and lactate dehydrogenase (LDH) enzyme kits were obtained from Bio-Rad (UK). The p53, bax and bcl-2 monoclonal antibody, and HRP-conjugated goat anti-rabbit IgG were purchased from Sigma-Aldrich (St. Louis, MO 63178 USA).

### Preparation of plant extract

Ir was obtained from National Research Institute for Sowa Rigpa (Amchi) Research Centre, Leh-Ladakh, India. Ir (2 kg) were cut into small pieces, shade dried for 7 days. A closed container was used in which Ir was taken and treated in *n*-hexane for about 2 days with infrequent shaking (Mohan and Gupta 2017). Marc was pressed after the n-hexane was strained off and kept for 4 days with infrequent shaking in a hydroalcoholic mixture. Until the formation of brown colored paste, the solution was filtered and concentrated. Two liters of n-hexane and methanol was used.

### Gas chromatography-mass spectrometry (GC/MS) analysis

Ir extracts were analyzed using gas chromatography-mass spectrometry (GC/MS, Thermo Fisher Scientific Korea Ltd. Seoul 06177, Korea). Compound identification was based on the retention time values and reported literature for authentic compounds (Kalachaveedu et al. 2017).

### Liquid chromatography-mass spectrometry (HPLC/MS) analysis

Component analysis of Ir extracts was performed by liquid chromatography-mass spectrometry (HPLC/MS, Thermo Fisher Scientific Korea Ltd. Seoul 06177, Korea) analysis (Agilent 6500 Series) (So Hyun et al. 2017).

### Animals

Healthy male albino Wistar strain rats were obtained from the animal house, Shangai, China, weighing (180–200 g) was selected for the present study. Animals kept in polypropylene cages, at temperature 25 ± 0.5 °C, relative humidity 60 ± 5% and a photoperiod of 12 h/day. All the animals were handled according to internationally accepted ethical procedures. Ethical approval was obtained from the Ethics Committee of Wenzhou Medical University (Approval No. 201308807).

### Induction of hepatic I/R

The foods were removed, and animals fasted before the experiment. Ketamine hydrochloride (100 mg/kg) and xylazine (10 mg/kg) were used to anesthetize animals. Ischemia was induced by clamping the hepatic portal triad. Bulldog clamp was used to clamp the hepatic portal train for 40 min which in turn produced Ischemia. Repercussion was generated through unclamping the triad for 40 min (Manipuri et al. 2013).

### Experimental group

Animals were divided into four each containing six. Group I: sham, group II: control, group III: I/R + Ir (100 mg/kg) and group IV: I/R + Ir (200 mg/kg). The oral gauge was used for drug administration for the 15 consecutive days.

### Collection of blood and liver

Blood was collected from all animals through cardiac puncture. Animals were sacrificed by decapitation, and liver tissue was surgically removed and place ice-cold saline and kept at − 20 °C for the further experiments.

### Determination of total phenolic contents

The total phenolic contents were determined with use of Folin–Ciocalteu method. Experimental data were expressed as caffeic acid equivalents per mg of dry extract weight (Faten et al. 2014). The total anthocyanin was measured with use of the pH differential method. Experimental data are expressed as mg of dry weight (Shoib and Shahid 2015).

### DPPH scavenging activity

α, α-diphenyl-β-picrylhydrazyl (DPPH) reduction was measured by the standard method, and the experimental results are expressed as μg of extract dry weight (Cavin et al. 1998).

### ABTS scavenging activity

2,2′-Azino-bis(3-ethylbenzothiazoline-6-sulphonic acid) (ABTS) scavenging activity was measured by the well-known standard method, and the experimental data are expressed as mg of dry extract weight (Re et al. 1999).

### Ferric reducing antioxidant power (FRAP)

The reducing ability of extract was determined with use of FRAP analysis, and it was determined by the standard method. Experimental data are expressed as μmol of extract dry weight (Benzie and Strain 1996).

### Determination of serum enzymes

ALT, AST, ALP, and LDH were determined in the serum by using kit (Span Diagnostics Ltd., India) method (Muthuviveganandavel et al. 2008).

### Histopathological and biochemical assays

Histopathological studies were conducted with sections (Kedee New, High Guality and Stable Rotary Microtome, Zhejiang Jinhua Kedi Instrumental Equipment Co., Ltd. Zhejiang, China) of liver fixed in formalin and staining was carried out with hydrated tissue sections in 5 μm with Hematoxylin and Eosin (H & E). The sections were observed under a light microscope (Muthuviveganandavel et al. 2008).

### Determination of TNF-α and IL-6 content

Tumor necrosis factor-alpha (TNF-α) and interleukin (IL-6) content were determined in the plasma. Enzyme-linked immune sorbent assay (ELISA) method was used to determine IL-6 and TNF-α in the plasma. Briefly, IL-6 and TNF-α present in the plasma to anti-IL-6 and anti-TNF-α monoclonal antibody adsorbed to the microwells. A biotin-conjugated monoclonal anti-IL-6 and anti-TNF-α antibody were incubated with IL-6 and TNF-α antibody. The unbound antibody has been removed through repeated washing with PBS. Then, streptavidin-HRP was incubated with biotin-conjugated anti-IL-6 and anti-TNF-α, and substrate HRP was added to samples. The resultant colored product was measured at 450 nm (Afshari et al. 2005).

### Western blot analysis

Cell homogenate was washed with PBS, and lysed with 10 mM Tris–HCl (pH 7.5), 100 mM NaCl, 1% NP-40, 50 mM NaF, 2 mM EDTA (pH 8.0), 10 μg/mL leupeptin, 1 mM PMSF and 10 μg/mL aprotinin. The protein which is present in the lysate was run on SDS-PAGE. PVDF membrane was used for transferring in the SDS-PAGE. TBST was used for the non-specific blocking proteins. The membrane probed for 12 h with an antibody against p53, Bax, and Bcl-2. Membranes were washed twice with TBST and incubated with HRP-conjugated goat anti-rabbit IgG (St. Louis, MO 63178 USA) for 60 min. The protein levels of p53, bax, and bcl-2 were determined by using enhanced chemiluminescence method (Muthuraman et al. 2014).

### Immunohistochemical analysis

Liver tissue was surgically removed from the rat animals following decapitation and rinsed in ice-cold normal saline. Paraformaldehyde was used for fixation of liver and dehydrated with ethanol. Then, tissues were embedded in paraffin wax and dewaxed and rehydrated before sectioning. Sections were made and incubated with mouse anti-p53, anti-bax and anti-bcl-2 (1:300, Abcam, USA) for overnight at 4 °C. After repeated washing with PBS, sections were incubated with HRP-conjugated secondary antibody at 37 °C for 60 min. Sections were counterstained with hematoxylin (Muthuraman and Srikumar 2009).

### Statistical analysis

All the experimental values are expressed as a mean ± standard error of the mean (SEM). The control and treated groups were compared using ANOVA (SPSS 15, Chicago, IL, USA). Furthermore, all the groups are compared using Student “t” test. A P < 0.05 was considered statistically significant.

## Results

The GC–MS analysis was used to get preliminary data on the composition of Ir extracts in the present study. The polarity of the solvents could affect the efficiency of extraction and activity of obtained compounds in the extracts. Ethyl acetate, ethanol, methanol, acetone, and water are most generally solvents for extraction. The compound obtained in the Ir extract is given in Table 1. HPLC/MS provides cost-effective tool for the identification of phenolic compounds. The chemical constituents are expressed on the dry weight basis. The compounds obtained in the Ir extracts are given in Table 2.

**Table 1:** Retention time (Rt) of compounds identified in the extract of Ir

No	Constituent	Retention time (min)	Identification
1	2-Methoxyethanol	15.241	Ref, Lib
2	Nonane, 3-methyl-5-propyl-methyl benzoate	16.013	Ref, Lib
3	Alpha-muurolene	19.435	Ref, Lib
4	2-Phenyl-2-propanol	21.618	Ref, Lib
5	Benzene, 1-(1,5-dimethyl-4-hexenyl)-4-methyl-1-butanol	22.624	Ref, Lib
6	Hexanal dimethyl acetal	23.958	Ref, Lib
7	2-Methoxyethanol	24.451	Ref, Lib
8	1-Pentanol	25.223	Ref, Lib
9	Propionitrile, 2-(3-fluorophenylhydrazono)-3-imino-3-(4-morpholyl)- tetradecane	36.521	Ref, Lib
10	1-Hexanol, 2-ethyl	43.536	Ref, Lib
11	2-Propenoic acid, 2-methyl-cyclohexyl ester	44.321	Ref, Lib
12	Beta elemene	51.612	Ref, Lib
13	Benzaldehyde	46.622	Ref, Lib
14	Nonane, 3-Methyl-5-propylnonane	50.776	Ref, Lib
15	Methyl benzoate	53.745	Ref, Lib
16	Benzoic acid	52.206	Ref, Lib
17	Acetophenone	56.414	Ref, Lib
18	Alpha-amorphene	56.494	Ref, Lib
19	Alpha-muurolene	55.394	Ref, Lib
20	2-Phenyl-2-propanol	58.333	Ref, Lib
21	Alpha-selinene	59.103	Ref, Lib
22	Beta-selinene	61.111	Ref, Lib
23	Azulene	62.495	Ref, Lib

*GC/MS* comparison with GC/MS standard, *Ref* comparison with GC and MS literature values

**Table 2:** The compounds obtained in the Ir extracts

S.No	Name of the constituents	Quantity (mg/100 g)
1	3,5-Dihydroxybenzoic acid-*O*-glucosyl-xyloside	13.9
2	Chlorogenic acid	33.2
3	Catechin gallate	22.5
4	Caffeic acid	93.72
5	Alantolactone	42.42
6	Kaempherol-7-*O*-dipentoside	42.9
7	Galloyl-caffeoylhexose	11.9
8	Quercetin-3-*O*-beta-glucopyranoside	51.1
9	Dicaffeoyl quinic acid	70.3
10	9-*O*-beta-d-glucopyranosyl-9-hydroxythymol	19.2
11	5-*O*-feruloylquinic acid	29.8
12	Dihydrocaffeic acid derivative	13.2
13	Epicatechin	22.7
14	6″-O-malonyl genistein	1.6

**Fig. 1:**
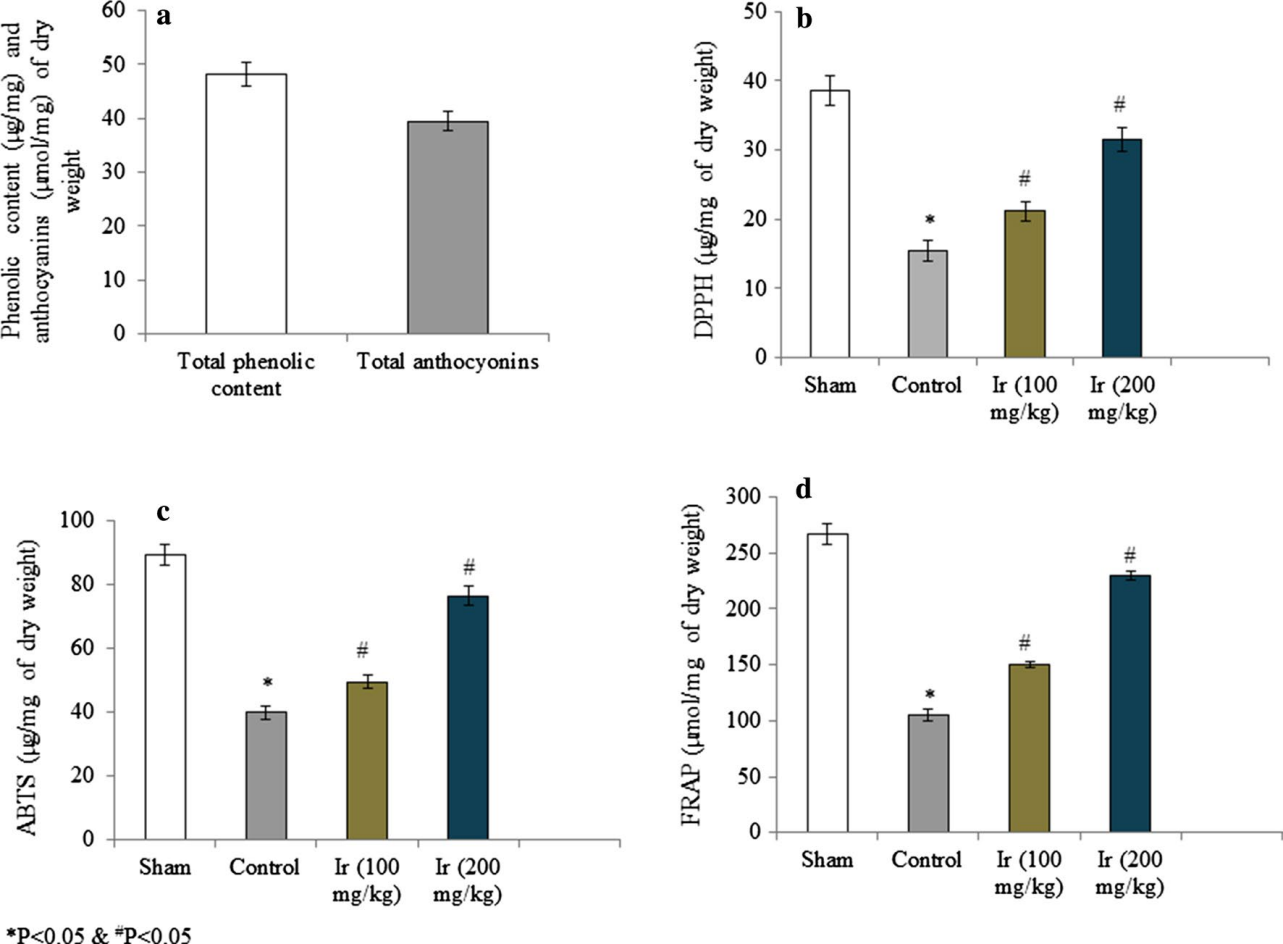
Total phenolic and anthocyanins content in the extract of Ir (**a**). DPPH activity in control and treated rats (**b**). ABTS activity in control and treated rats (**c**). FRAP activity in control and treated rats (**d**). N = 6, *P < 0.05 vs. control group, ^#^ *P* < 0.05 vs. treated groups

The total phenolic and anthocyanins contents were determined in the extract of Ir. Total phenolic and anthocyanins contents were 48.26 µg/mg and 39.5 µmol/mg of dry weight respectively (Fig. 1a). DPPH scavenging activity was 38.6 and 15.4 µg/mg of dry weight in the sham and control group respectively. Treatment of rats with extracts of Ir significantly improved DPPH scavenging activity. DPPH scavenging activity was 21.2 and 31.5 µg/mg of dry weight in the group III and IV respectively (Fig. 1b, P < 0.05). ABTS scavenging activity was 89.3 and 39.89 µg/mg of dry weight in the sham and control group respectively. Treatment of rats with extracts of Ir significantly improved ABTS scavenging activity to 49.5 and 76.48 µg/mg of dry weight in the group III and group IV respectively (Fig. 1c, P < 0.05). Ferric reducing antioxidant power was 266.6 and 105.34 µmol/mg of dry weight in the sham and control group respectively. Treatment of rats with extracts of Ir significantly improved FRAP to 149.61 and 229.81 µmol/mg of dry weight in the group III and group IV respectively (Fig. 1d, P < 0.05).

**Fig. 2:**
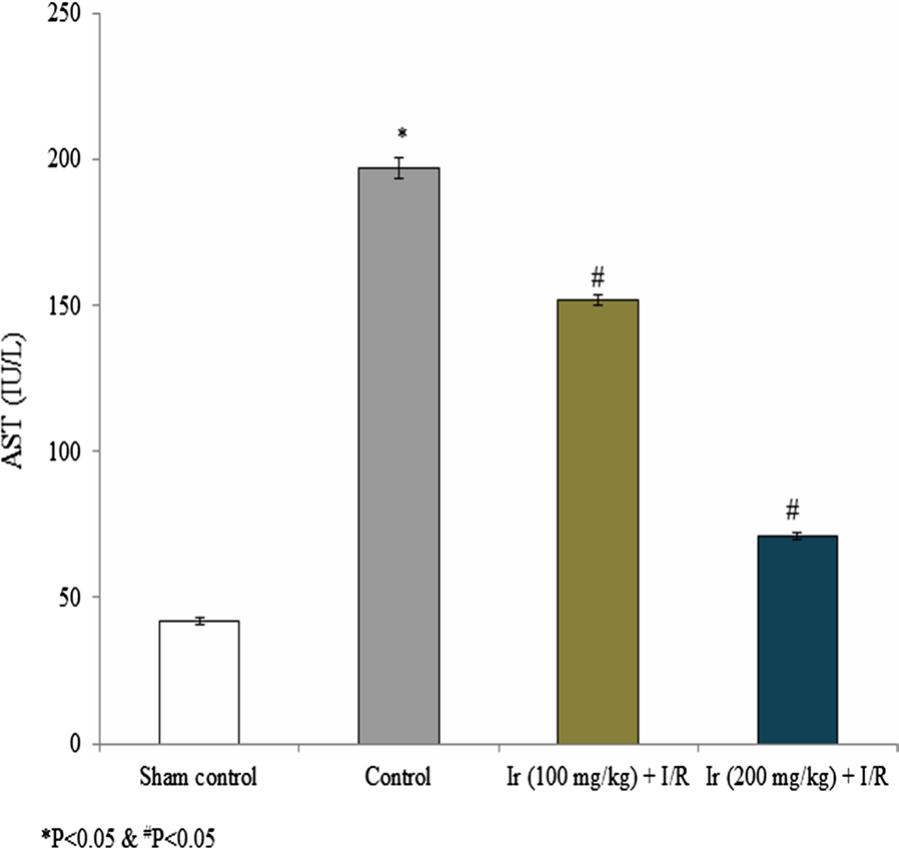
Ir attenuated serum AST enzyme level. Values are expressed IU/L. Results are shown mean with SEM. N = 6, *P < 0.05 vs. control group, ^#^ *P* < 0.05 vs. treated groups

**Fig. 3:**
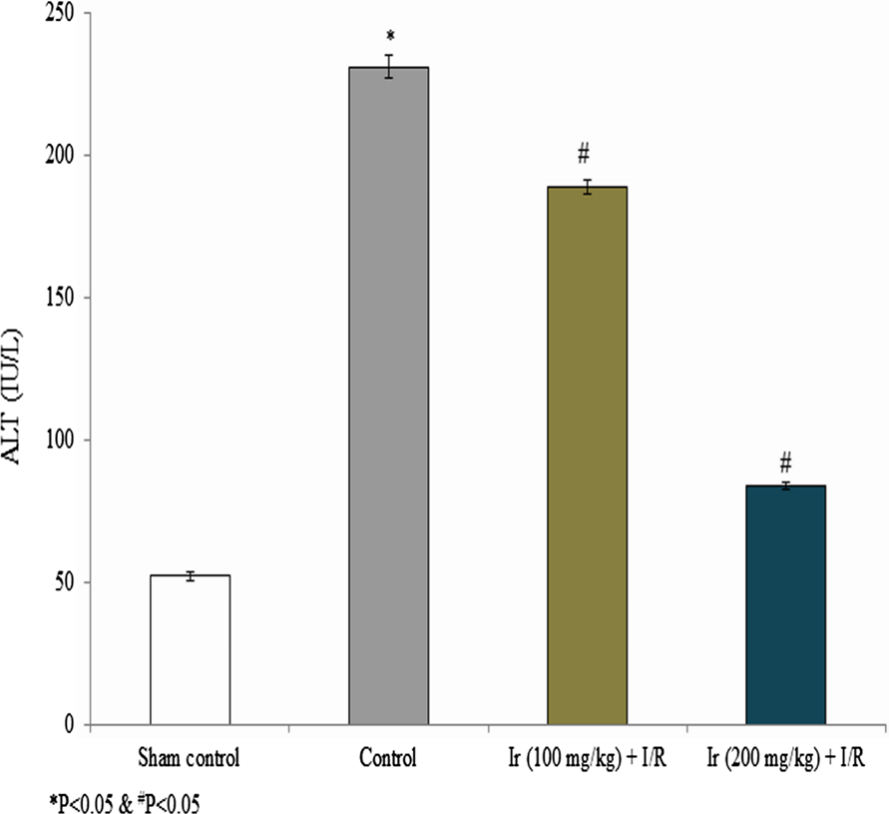
Ir attenuated serum ALT enzyme level. Values are expressed IU/L. Results are shown mean with SEM. N = 6, *P < 0.05 vs. control group, ^#^ *P* < 0.05 vs. treated groups

**Fig. 4:**
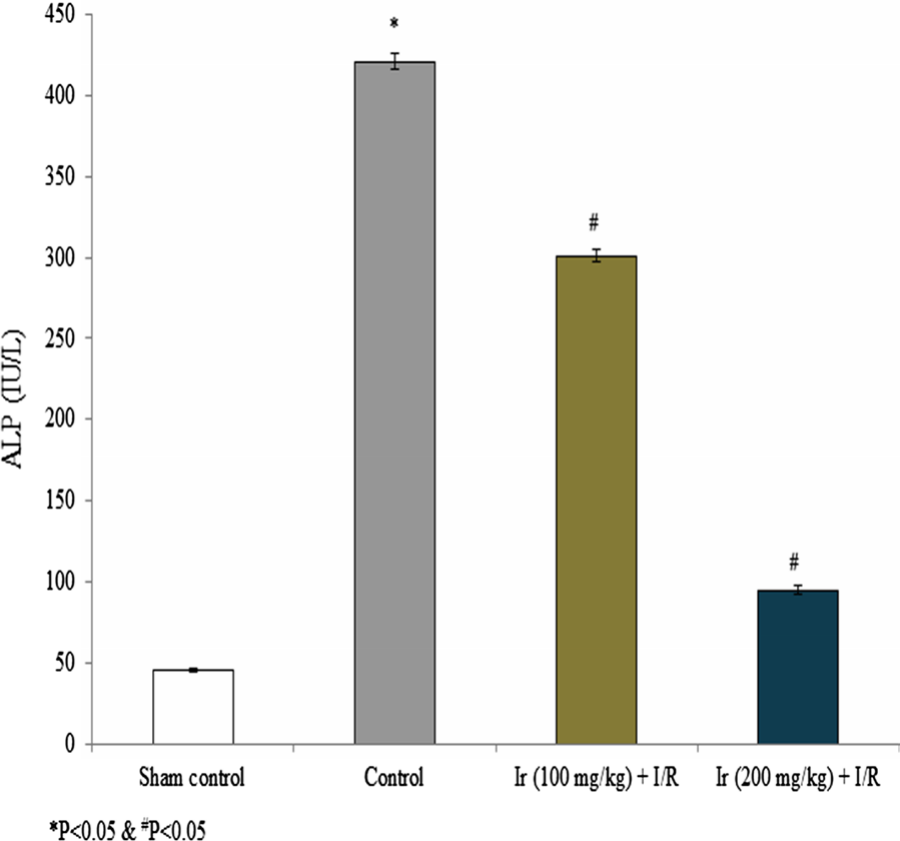
Ir attenuated serum ALP enzyme level. Values are expressed IU/L. Results are shown mean with SEM. N = 6, *P < 0.05 vs. control group, ^#^ *P* < 0.05 vs. treated groups

**Fig. 5:**
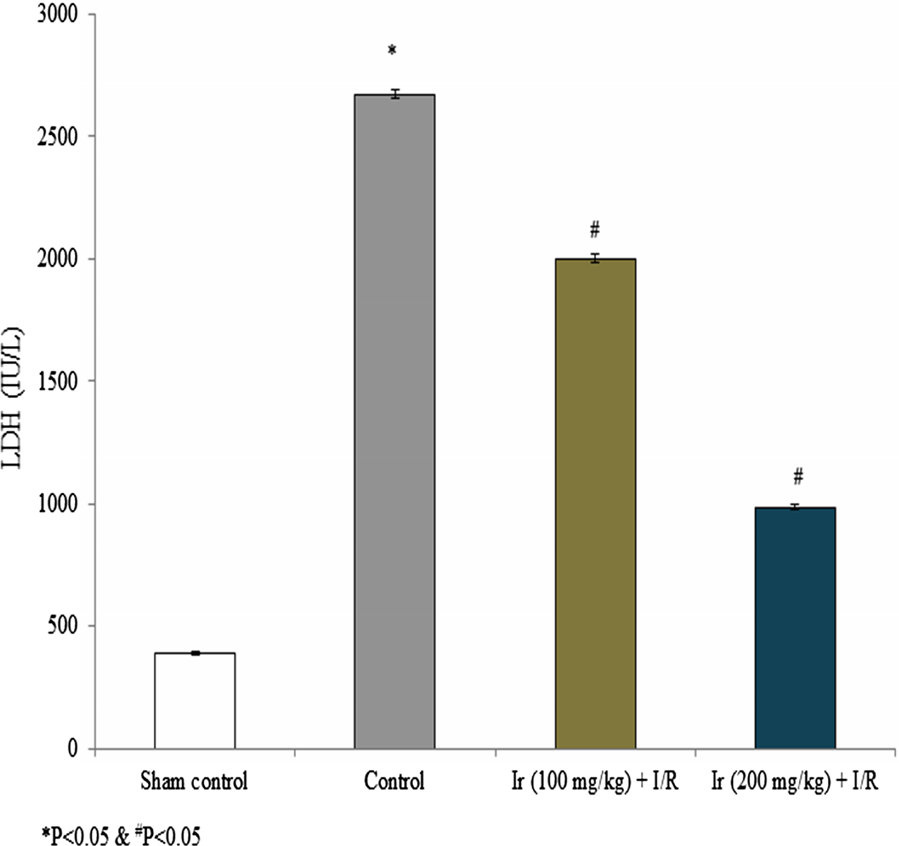
Ir attenuated serum LDH enzyme level. Values are expressed IU/L. Results are shown mean with SEM. N = 6, *P < 0.05 vs. control group, ^#^ *P* < 0.05 vs. treated groups

**Fig. 6:**
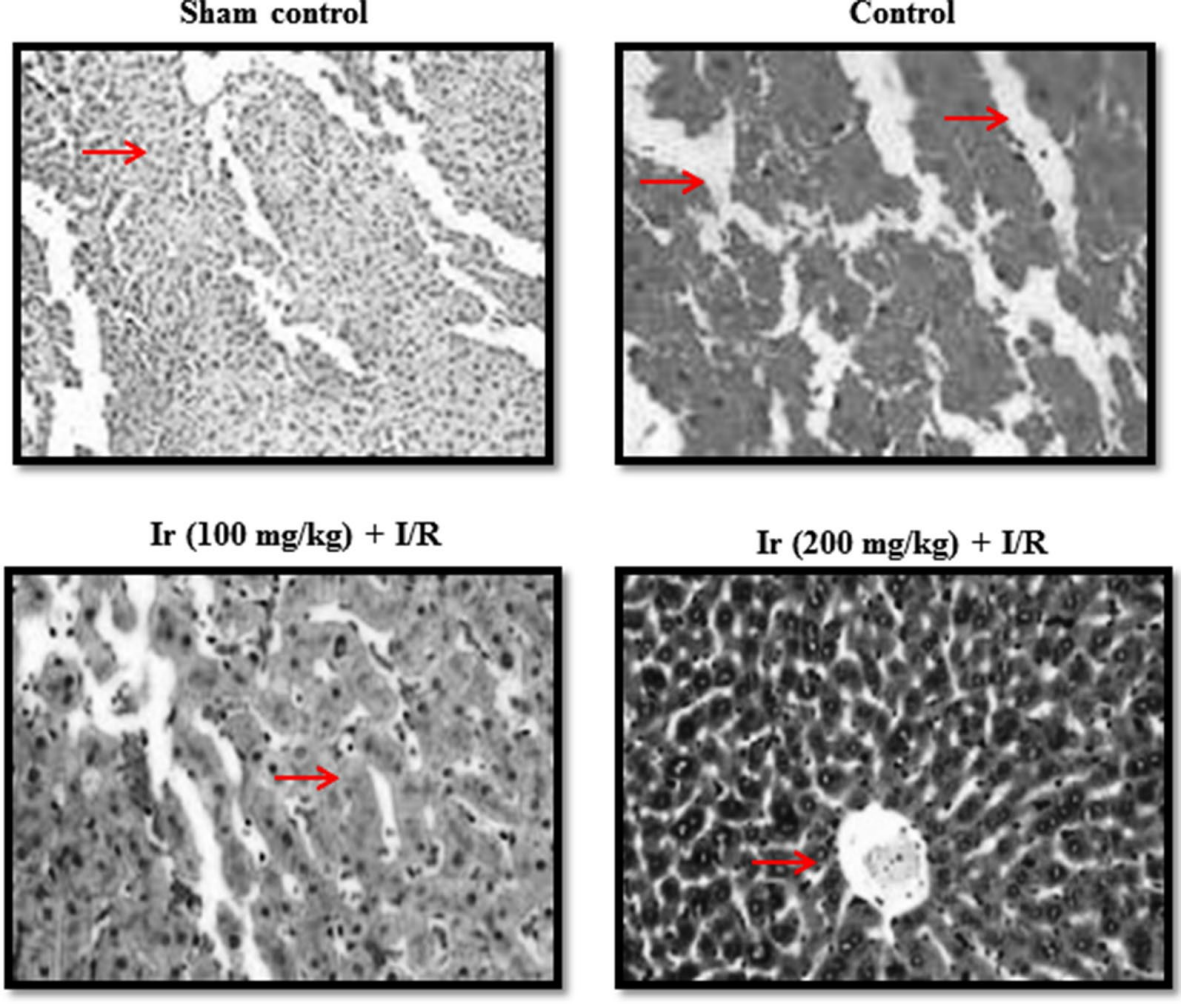
Ir attenuated altered cell morphology. Normal cellular architecture, and no congestion and necrosis (group I). Inflammation, congestion, and necrosis (group II). Attenuated inflammation, congestion, and necrosis (group III and IV). The representative images were obtained from six independent experiments

AST, ALT, ALP and LDH levels were reduced following treatment compared to the control. These serum enzymes were significantly reduced at higher concentration of Ir in this study. Treatment showed increased AST, ALT, ALP and LDH levels compared to the standard control, but lesser than model control which indicates that treatment had a significant effect on the reduction of these enzymes (Figs. 2, 3, 4, 5, P < 0.05). No pathology was found in the sham group. Liver histology showed the normal cellular architecture, and there was no congestion and necrosis. Liver cells were arranged in the cord. Several portal tracks were observed in the liver histology. Dilated sinusoids and veins were seen, as well as inflammation and necrosis was found in the control group (Fig. 6). However, the Ir treatment significantly reduced these abnormalities compared.

**Fig. 7:**
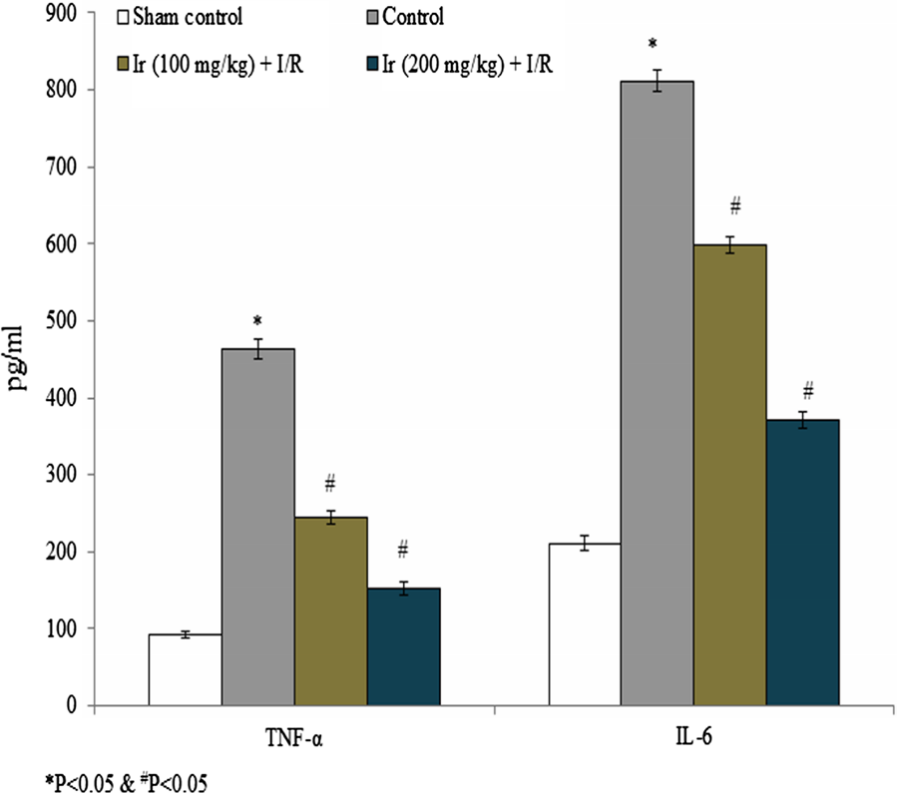
Ir attenuated serum IL-6 and TNF-α level. Values are expressed as pg/mL. The results are expressed as +SEM, N = 6, *P < 0.05 vs. control group, ^#^ *P* < 0.05 vs. treated group

IL-6 and TNF-α levels were determined to understand the effect of an extract of Ir on inflammation. IL-6 and TNF-α levels were significantly increased 284.36 and 397.85% in the control rats compared to the sham. However, the treatment of extracts of Ir significantly reduced the IL-6 level to 26.26 and 54.25% in group III and group IV respectively. The TNF-α concentration was reduced to 47.08 and 67.17% in group III and group IV respectively (Fig. 7, P < 0.05). Reduced level of cytokines revealed that extract of Ir possesses hepato-protective activity in hepatic ischemic/reperfusion injury in rats.

**Fig. 8:**
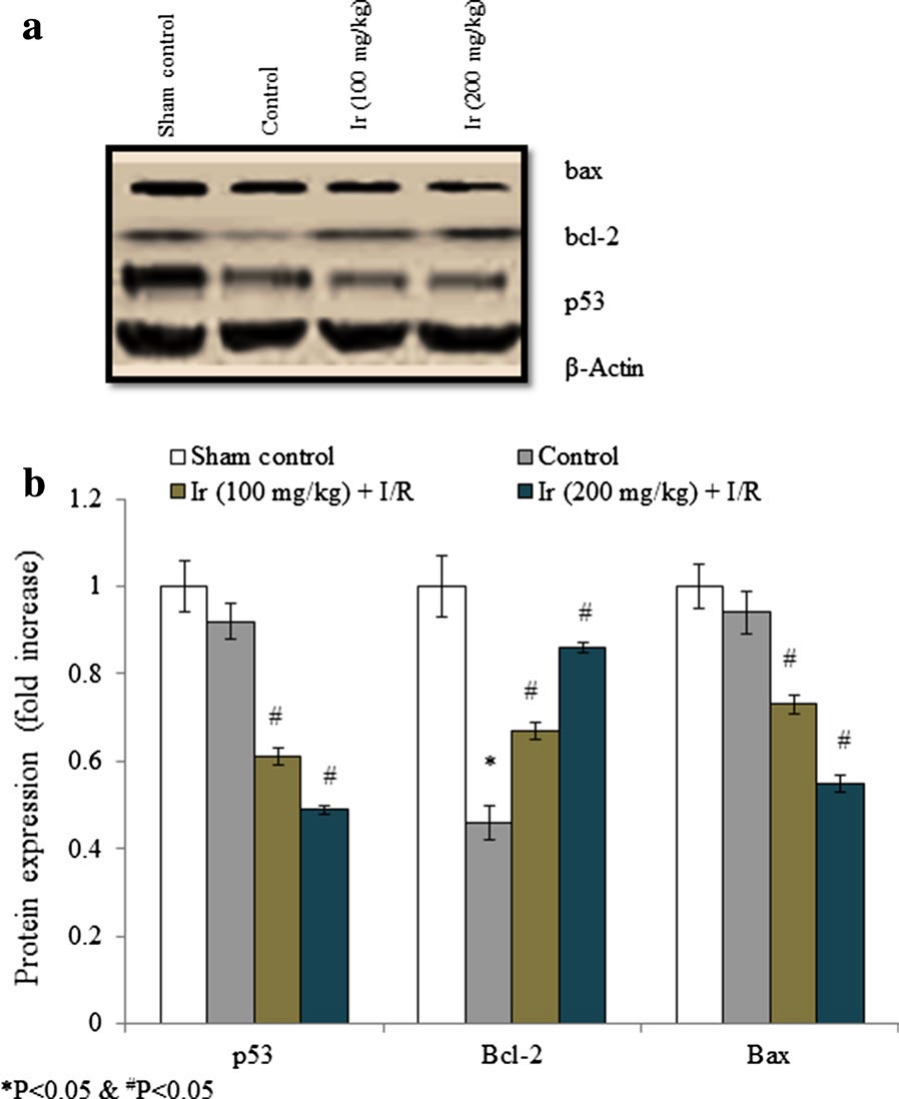
The effect of Ir on the protein expression of p53, bcl-2 and bax. The western blot (**a**) and presentative images of p53, bcl-2 and bax (**b**). The results are expressed as +SEM, N = 6, *P < 0.05 vs. control group, ^#^ *P* < 0.05 vs. treated groups

To understand the effect of Ir on protein expression of p54, bax and bcl-2, we carried out western blot analysis. Protein expression of p54, bax and bcl-2 were significantly altered compared to the control. The bcl-2 protein expression was reduced to 0.54 fold in control compared to the sham control. Ir treatment significantly increased bcl-2 expression 0.45 and 0.87 folds in group III and group IV respectively. The p53 protein expression was reduced to 0.08-fold in control compared to the sham control. Ir treatment significantly reduced p53 expression 0.34- and 0.47-folds in group III and group IV respectively. The bax protein expression was reduced to 0.04-fold in control compared to the sham control. Ir treatment significantly reduced bax expression 0.22- and 0.41-folds in group III and group IV respectively (Fig. 8, P < 0.05). Renormalization of cancer apoptotic gene expression revealed that the extract of Ir possesses hepato-protective activity in hepatic ischemic/reperfusion injury in rats.

**Fig. 9:**
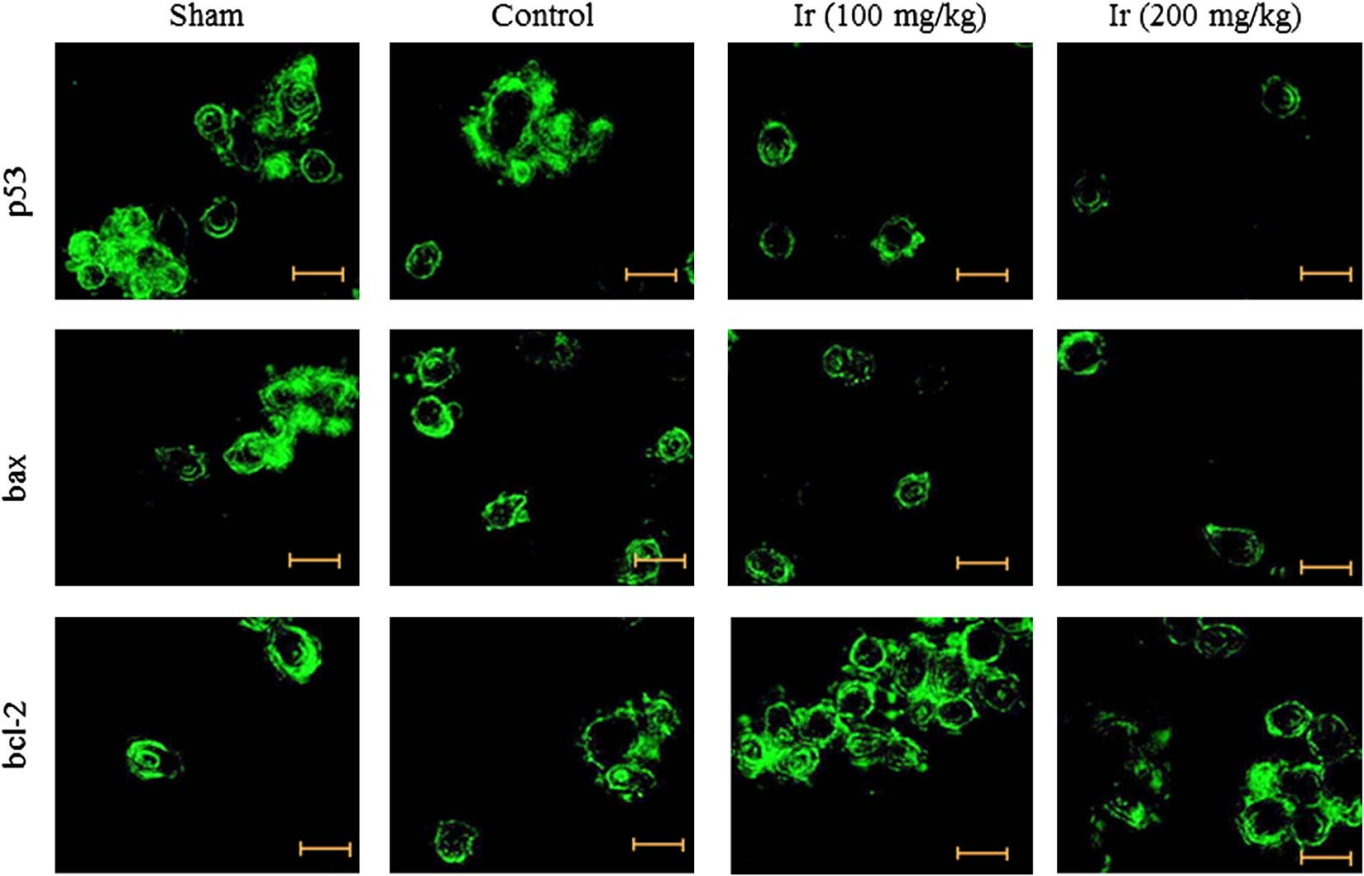
Immunofluorescence of p53, bcl-2 and bax. The representative images were obtained from six independent experiments. Scale bar is 60 µm

Immunohistochemistry revealed the effect of Ir on p53, bax and bcl-2 expression. The p53, bax and bcl-2 protein expression were reduced in control compared to the sham control. Ir treatment significantly reduced the p53 and bax expression compared to the control, whereas bcl-2 expression was dramatically increased compared to the control. The effect was found in a dose-dependent manner (Fig. 9).

## Discussion

The GC–MS and HPLC/MS analysis was used to get preliminary data on the composition of Ir extracts in the present study. The presence of alkaloids, phenols and flavonoids in the extract of Ir may induce directly or indirectly to neutralize the oxidants and activation of free radical scavenging system (Dinkova-Kostova 2008). Zheng and Wang (2001) have reported that the degree of flavonoid and polyphenol abundance contains positive correlation to its free radical scavenging and antioxidant potential. Our results agree with findings of Mohan and Gupta (2017) who have stated that the right antioxidant activity of extracts of Ir in ABTS and FRAP assays. Our results agreed with findings of Manipuri et al. (2013) who have reported that the reduced level of serum hepatic enzymes and renormalization of altered cellular morphology following treatment of Ir in hepatic I/R injury in rats.

To understand the effect of Ir on protein expression of p54, bax and bcl-2, we carried out western blot analysis. Protein expression of p54, bax and bcl-2 were significantly altered compared to the control. Renormalization of cancer apoptotic gene expression revealed that the extract of Ir possesses hepato-protective activity in hepatic ischemic/reperfusion injury in rats. Immunohistochemistry revealed the effect of Ir on p53, bax and bcl-2 expression. The p53, bax and bcl-2 protein expression were reduced in control compared to the sham control. Ir treatment significantly reduced the p53 and bax expression compared to the control, whereas bcl-2 expression was dramatically increased compared to the control. The effect was found in a dose-dependent manner. The medicinal property of Ir has been extensively studied in the ayurvedic system in rodents and human models (Miller 1998). Cardioprotective effect of Ir has been reported against isoproterenol-induced myocardial infarction (Ojha et al. 2011).

In summary, the administration of extracts of Ir significantly increased total phenolic content and free radical scavenging activity. Altered cellular morphology, cytokines and AST, ALT, ALP, and LDH were returned to near normal level. Also, the protein expression of p53, bax, and bcl-2 were also returned to near normal level. Taking all these data together, it is suggested that the extracts of Ir may be a potential therapeutic agent for providing several beneficial effects in hepatic I/R injury following orthotopic liver transplantation.

## Abbreviations

I/R: ischemia–reperfusion (I/R)
Ir: *Inula racemosa*
AST: aspartate aminotransferase
ALT: alanine aminotransferase
ALP: alkaline phosphatase
LDH: lactate dehydrogenase
DMSO: dimethyl sulfoxide
FRAP: ferric reducing antioxidant power
DPPH: α, α-diphenyl-β-picrylhydrazyl
ABTS: 2,2′-azino-bis(3-ethylbenzothiazoline-6-sulphonic acid)
TNF-α: tumor necrosis factor-alpha
IL6: interleukin (IL-6)
ELISA: enzyme-linked immune sorbent assay
PVDF: polyvinyl difluoride
SEM: standard error of the mean
